# Evaluation of prenatal changes in fetal cardiac morphology and function in maternal diabetes mellitus using a novel fetal speckle-tracking analysis: a prospective cohort study

**DOI:** 10.1186/s12947-021-00256-z

**Published:** 2021-06-30

**Authors:** Dong Wang, Caixia Liu, Xinyu Liu, Ying Zhang, Yu Wang

**Affiliations:** Department of Ultrasound, Shengjing Hospital of China Medical University, No. 36 Sanhao Street, Heping District, Shenyang, 110004 China

**Keywords:** Gestational diabetes mellitus, Fetal cardiac morphology, Cardiac function, Echocardiography, Fetal HQ

## Abstract

**Background:**

Due to metabolic changes in the second trimester and the increasing number of pregnant women with obesity and advanced maternal age, the incidence of gestational diabetes mellitus (GDM) remains high. This study aimed to evaluate the effects of GDM on fetal cardiac morphology and function, and to determine whether these changes increase with increasing estimated fetal weight (EFW).

**Methods:**

Fifty-eight women with GDM (GDM group) and 58 women with a healthy pregnancy (control group) were included in this prospective observational cohort study. Each group included subgroups of 31 pregnant women with a gestational age between 24^+0^ weeks and 27^+6^ weeks as well as 27 pregnant women with a gestational age between 28^+0^ weeks and 40^+0^ weeks. For all fetuses, a cine of 2–3 s in the four-chamber view was obtained, and online speckle-tracking analysis was performed using the GE Automatic Fetal Heart Assessment Tool (fetal HQ; General Electric Healthcare Ultrasound, Zipf, Austria) to measure the global sphericity index (GSI), global longitudinal strain (GLS), fractional area change (FAC), 24-segment sphericity index (SI), and 24-segment end-diastolic diameter of the left ventricle (LV) and right ventricle (RV). Data were analyzed using the independent t-test and Wilcoxon rank-sum test, as applicable.

**Results:**

The GDM group (mean HbA1c value was 5.3 ± 0.57 mmol/L) showed a lower GSI value than the control group (1.21 vs. 1.27, *P* = 0.000), which indicated a rounder shape of the heart. In addition, fetuses in the GDM group demonstrated significant impairment in cardiac function compared to those in the control group (LV-GLS: -18.26% vs. -22.70%, RV-GLS: -18.52% vs. -22.74%, LV-FAC: 35.30% vs. 42.36%, RV-FAC: 30.89% vs. 36.80%; *P* = 0.000 for all). Subgroup analyses according to gestational age (24^+0^–27^+6^ weeks and 28^+0^–40^+0^ weeks) showed that the statistical differences were retained between the GDM and control groups in each subgroup.

**Conclusions:**

Fetuses of women with GDM present with signs of biventricular systolic dysfunction according to deformation analysis using fetal HQ. Additionally, the heart had a rounder shape in the GDM group than in the control group. This study showed that fetal HQ can be used to assess fetal cardiac morphology and function easily and quickly, and the effects of GDM on fetal cardiac morphology and function appeared from the second trimester. Thus, whether earlier and stricter clinical intervention was necessary remained to be further studied. Furthermore, future studies will need to supplement the effects of blood glucose levels on GLS, FAC, GSI, and 24-segment SI. Additionally, the long-term follow-up after birth should also be improved to observe the influence of changes in the indicators on the prognosis.

**Supplementary Information:**

The online version contains supplementary material available at 10.1186/s12947-021-00256-z.

## Background

Hyperglycemia in pregnancy can be classified as either pre-gestational diabetes or gestational diabetes mellitus (GDM). GDM refers to diabetes that appears for the first time during pregnancy [1, 2]. Due to metabolic changes in the second trimester and the increasing number of pregnant women with obesity and advanced maternal age, the incidence of GDM remains high.

Hyperglycemia not only affects fetal development but also has teratogenic effects on fetal hearts. According to research studies on animals, fetuses exposed to a high maternal blood glucose level can show myocardial remodeling [3, 4]. Besides structural malformations of the heart, the most common heart disease caused by GDM is hypertrophic cardiomyopathy. However, impairment of fetal cardiac function may occur before myocardial thickening [5]. In the past few years, ultrasonography has been used to assess changes in ventricular function. In addition to traditional Doppler and tissue Doppler ultrasonography, speckle-tracking echocardiography (STE) has become a popular modality in recent years, especially for assessing adult cardiac function. The speckle-tracking mode used to assess adult cardiac function was chosen by many authors to assess changes in fetal ventricular segmental function [6, 7]. The causes and mechanisms of changes in ventricular function in fetuses are not the same as those in adults.

The current study used a novel technology, the GE Automatic Fetal Heart Assessment Tool (fetal HQ; General Electric Healthcare Ultrasound, Zipf, Austria), to assess changes in fetal cardiac morphology and ventricular function. This was the first application of fetal HQ in the evaluation of fetal cardiac function of mothers with GDM. Therefore, we attempted to explore the feasibility and advantages of fetal HQ. This study aimed to evaluate the effects of GDM on the morphology and function of the fetal heart during the second and third trimesters and to determine whether the degree of change increases with increasing estimated fetal weight (EFW). We hypothesized that the fetuses of mothers with GDM would present with signs of cardiac dysfunction and an abnormal cardiac shape, and that fetal HQ could be a simple tool used to generate reproducible results for assessing cardiac morphology and function in this condition.

## Methods

### Study population

This prospective observational cohort study included 58 fetuses of mothers with GDM as the case group and another 58 matched fetuses of mothers with normal pregnancies as the control group. Each group was further divided into two subgroups according to gestational age: 31 pregnant women with a gestational age of 24^+0^–27^+6^ weeks, and 27 pregnant women with a gestational age of 28^+0^–40^+0^ weeks. Gestational age was determined based on the last menstrual period and EFW was based the measurement of the fetal biparietal diameter (BPD), head circumference (HC), abdominal circumference (AC), and femur length (FL). Cases and controls were recruited from October 2019 to November 2020 at the Department of Ultrasound, Shengjing Hospital of China Medical University, Shenyang, China.

The diagnosis of GDM was followed by a one-step 75-g oral glucose tolerance test (OGTT), based on the International Association of Diabetes and Pregnancy Study Groups criteria, between 24 and 28 gestational weeks. The OGTT included the glucose level measurements at three phases: fasting plasma glucose, 1-h plasma glucose, and 2-h plasma glucose. A diagnosis of GDM was made if the corresponding value was ≥ 5.1 mmol/L (92 mg/dL), 10.0 mmol/L (180 mg/dL), and 8.5 mmol/L (153 mg/dL), respectively [8].

### Ethics approval and consent to participate

This study was approved by the Ethics Committee of Shengjing Hospital of China Medical University. Written informed consent was obtained from the patients for the publication of clinical details, clinical images, and videos.

### Inclusion and exclusion criteria

The inclusion criteria for the GDM group were as follows: singleton pregnancies of mothers aged 18 years or older, positive diagnosis for GDM at Shengjing Hospital, and gestational age of 24–40 weeks. The inclusion criterion for the control group was normal fetuses matched with the GDM fetuses by gestational age. The exclusion criteria for the GDM and the control groups were as follows: fetuses with structural/chromosomal anomalies; a persistently high fetal heart rate (FHR) (> 160 beats per minute); maternal smoking during pregnancy; twin or multiple pregnancies; inadequate echocardiographic images; any maternal disease including chronic hypertension, diabetes mellitus before pregnancy, human immunodeficiency virus or hepatitis infection, thyroid disease, or other diseases that may affect fetal heart morphology and function; and fetuses with congenital heart disease, arrhythmia, or malformation involving other organs. Small- or large-for-gestational age status was considered as an additional exclusion criterion and was defined as a birth weight below the 10^th^ or above the 90^th^ percentile, respectively, according to the 2015 Chinese sex- and gestational age-specific birth weight standards [9]; refusal of the mother to provide written informed consent was also an exclusion criterion. The control group underwent the same study protocol as the GDM group.

### Maternal characteristics

Maternal age, height, weight before pregnancy, method of conception (natural or conception using ovulation induction drugs or assisted reproductive technologies), number of pregnancies or births, smoking status during pregnancy, and family history of diabetes mellitus were recorded by two operators (CX L and XY L), together with the OGTT results and glycosylated hemoglobin (HbA1c) levels of women in the GDM group.

### Fetal cardiac morphology and function

Prenatal ultrasound was performed using the GE Voluson E10 Ultrasound System (General Electric Healthcare Ultrasound, Zipf, Austria) equipped with a 2–9 MHz wide-band convex probe (C2-9-D).

### Basic evaluation of fetuses

The overall condition of the fetuses was evaluated first. The values of fetal BPD, HC, AC, and FL were measured and recorded to estimate the fetal weights and correct the gestational ages. Basic Doppler evaluation included measurements of the resistance index and pulsatility index of the umbilical artery and middle cerebral artery, as well as the cerebroplacental ratio and FHR.

### Imaging acquisition in the four-chamber view

Every candidate had an optimal four-chamber view (4CV) for analysis. The orientation of the 4CV will change with variability in the fetal position. Still, not all 4CVs in each direction with different apex points can be analyzed by a physician. An ultrasonic beam perpendicular to or intersecting it with the interventricular septum is conducive to analysis. Therefore, we gave preference to those 4CVs in which the ultrasound beam crosses the cardiac interventricular septum perpendicularly or obliquely. And those 4CVs in which the interventricular septum of the heart parallel to the ultrasound beam were excluded. The acquisition of a dynamic image for an optimal 4CV needs to be completed when the mother is holding her breath and the fetus is not moving, with the frame rate set at > 80 frames/s [10], which can be adjusted by changing the width, depth, focus, or transducer of the narrow sector. Two or three seconds are enough for acquiring the image, as a longer time will increase the processing load of the machine and more importantly, cause blurring of the cardiac cycle, resulting in increased deviation. The fetal image in the 4CV was obtained by an experienced echocardiographer (DW) and reviewed by the senior author (YZ), neither of whom were aware of the grouping scheme and ultrasound diagnosis (although they knew the aims of the study).

### Measurements of the global heart size and shape in the four-chamber view

**Fig. 1:**
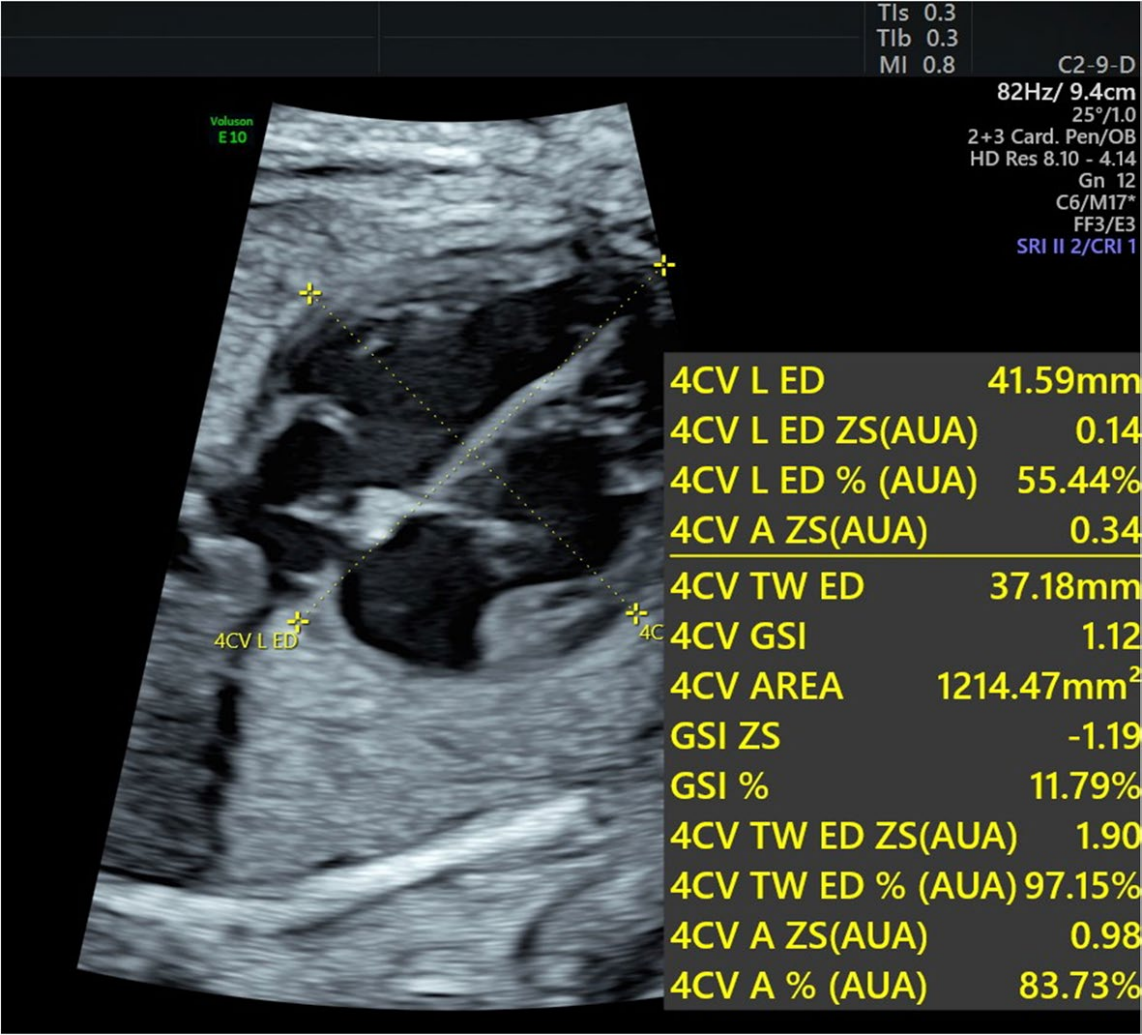
Measurement of the fetal cardiac GSI of gestational diabetes mothers at 32 gestational weeks. A longitudinal line is drawn from the apex to the base of the cardiac outer edge and a transverse line is drawn from the sidewall of the LV to the sidewall of the RV at the end of diastole. The GSI can be obtained by dividing the end-diastolic basal–apical length by the end-diastolic transverse width. GSI: global sphericity index; LV: left ventricle; RV: right ventricle

Fetal HQ combining TomTec analysis software and Professor Devore’s graphs, Z-scores, and centiles can be used on the new version of the GE Voluson E10 (General Electric Healthcare Ultrasound, Zipf, Austria) Ultrasound System. The tool first obtains measurements of the global heart size and shape in the 4CV. To start, we selected an optimal dynamic image of the 4CV and then clicked the “measure” button of the fetal HQ tool. In the operation interface of fetal HQ, the end-diastolic basal–apical length and the end-diastolic transverse width were measured in the longest dimension according to the prompts. The global sphericity index (GSI) was obtained by dividing the end-diastolic basal–apical length by the end-diastolic transverse width (Fig. 1).

### Evaluation of ventricular shape and contractility

**Fig. 2:**
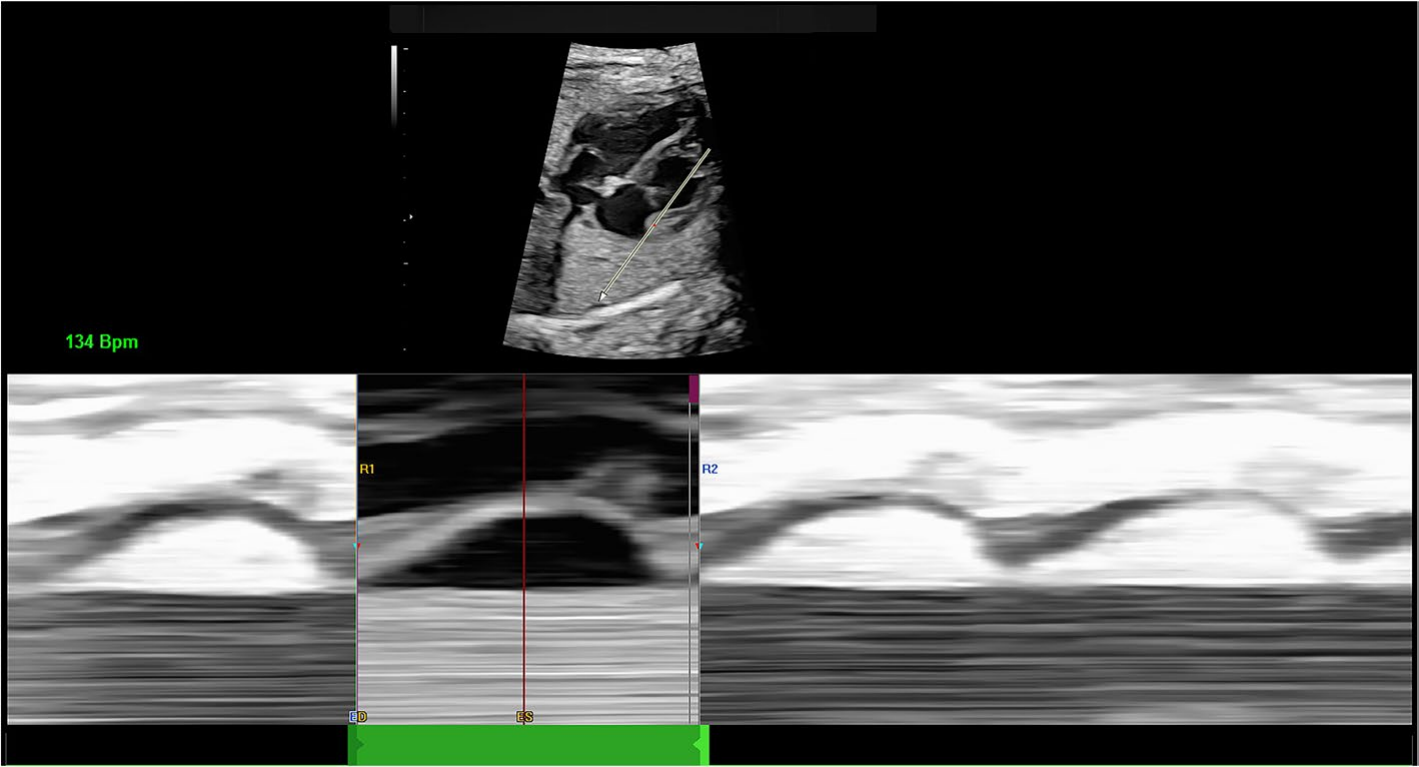
Determination of the cardiac cycle. This figure shows the determination of the right ventricular cardiac cycle, including the end-diastole and end-systole

First, we determined a cardiac cycle using M-mode. We drew a line from the apex to the base or across both ventricles with the trackball and set keys. We used the line through the apex of the right ventricle (RV) to the root of the tricuspid annulus to identify two subsequent end-diastolic periods as the cardiac cycle of the RV, and the same for the cardiac cycle of the left ventricle (LV). Clear M-mode tracking of end-systole and end-diastole is the foundation for an accurate assessment. It is worth noting that in adults, we define the start of ventricular end-diastole from the start of the Q wave on an electrocardiogram. For fetal hearts, we defined the cardiac cycle according to M-mode tracking at the root of the atrioventricular annulus and the open or closed state of atrioventricular valves, setting the highest point on the M-mode tracking corresponding to the first frame before the atrioventricular valve closed as end-diastole and the lowest point on the M-mode tracking corresponding to the first frame before the atrioventricular valve opened as end-systole. Moreover, we defined the left and right ventricular cardiac cycles separately if left ventricular movement did not synchronize with right ventricular movement (Fig. 2).

**Fig. 3:**
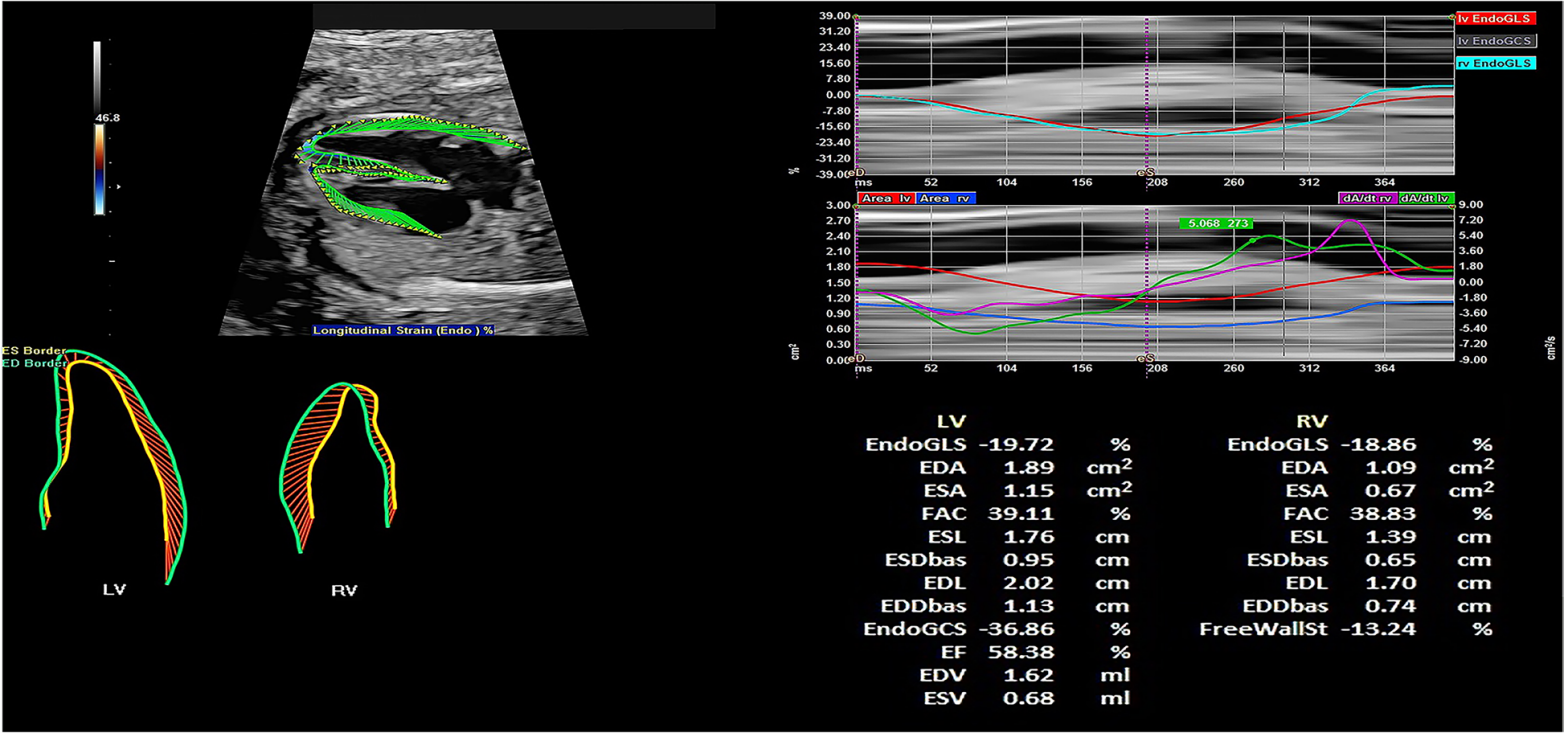
The results of fetal cardiac function in the GDM group. The GLS and FAC of the LV and RV in the GDM group are shown at the picture. GLS: global longitudinal strain; FAC: fractional area change; GDM: gestational diabetes mellitus; LV: left ventricle; RV: right ventricle

The systolic endocardial borders of each ventricle were automatically traced by the selection of three points in the pictogram containing the junction of the lateral wall annulus, the apex, and the junction of the septal wall annulus. Then, the diastolic endocardial borders of each ventricle were automatically tracked. If necessary, we modified the points of the curves generated by the software so that they were well matched with the endocardial borders. Then, we obtained the interface report, from which we collected data on the global longitudinal strain (GLS), fractional area change (FAC), 24-segment sphericity index (SI) (scores: base of the heart, 1; apex, 24), and 24-segment end-diastolic diameter of the LV and RV (See Additional file 1: Video and Fig. 3).

### Statistical analysis

Statistical Product and Service Solutions version 23.0 (IBM Corp.) was used to perform the statistical analyses. Normally distributed continuous variables are presented as the mean ± standard deviation (SD), and variables that did not follow a normal distribution are presented as medians. The distribution of continuous variables was graphically evaluated by histograms and quantile–quantile plots. An independent t-test was used to analyze normally distributed continuous data, and the Wilcoxon rank-sum test was used to analyze skewed data. Analysis was further adjusted for maternal age, BMI and EFW. The independent associations between the indicators and traditional cardiovascular risk factors such as maternal age, BMI, FHR and EFW were evaluated by Pearson correlation analysis. A *P*-value < 0.05 was considered statistically significant.

Although the echocardiographer knew the purpose of the study, they were blinded to the grouping scheme and ultrasound diagnosis. To evaluate intra/interobserver variabilities, speckle-tracking analysis was performed on 15 randomly selected cases by a second assessor (YW) and by the first echocardiographer (DW) after a month’s interval, respectively. Bland–Altman plots and intra-class correlation coefficients (ICC) were derived to assess the inter-observer and intra-observer variability. ICC values > 0.75 were suggestive of good reliability and > 0.9 of excellent reliability.

## Results

One hundred and sixteen pregnant women from Liaoning Province, China, were recruited in the study and five were excluded from final analysis because of inadequate echo images. There were no apparent differences between the groups in mean gestational age, EFW and FHR at investigation. However, women with GDM were older and had a higher body mass index (BMI) than the controls. And the mean value of HbA1c in the GDM group was 5.3 ± 0.57 mmol/L(Table 1). Besides, Fetal HQ was used to analyze the cardiac changes in the shape and function. And the mean analysis time using Fetal HQ was 6.2 ± 1.8 min.

**Table 1:** Baseline maternal and fetal characteristics of GDM group and control group

Characteristics	GDM group (mean ± SD)			Control group (mean ± SD)			*P*-value		
	24^+0^–27^+6^ weeks (*n* = 31)	28^+0^–40^+0^ weeks (*n* = 27)	Total (*n* = 58)	24^+0^–27^+6^ weeks (*n* = 31)	28^+0^–40^+0^ weeks (*n* = 27)	Total (*n* = 58)	P1	P2	P3
Maternal age (years)	31.1 ± 3.7	33.6 ± 3.1	32.3 ± 3.6	31.7 ± 3.1	29.8 ± 3.8	30.8 ± 3.5	0.456	0.000	0.033
BMI (Kg/m2)	25.0 ± 4.6	25.1 ± 4.2	25.0 ± 4.4	22.2 ± 3.2	22.7 ± 3.1	22.4 ± 3.1	0.008	0.020	0.000
Gestational age (weeks)	25.7 ± 1.4	30.7 ± 1.8	28.0 ± 2.9	25.8 ± 1.2	30.7 ± 2.5	28.1 ± 3.1	0.199	0.788	0.750
FHR (bpm)	148.26 ± 8.1	146.00 ± 8.47	147.12 ± 8.36	147.26 ± 6.2	144.11 ± 7.03	145.68 ± 7.09	0.586	0.377	0.315
EFW (Kg)	0.89 ± 0.18	1.70 ± 0.34	1.28 ± 0.49	0.90 ± 0.16	1.73 ± 0.55	1.29 ± 0.57	0.817	0.801	0.946
HbA1c (mmol/L)			5.3 ± 0.57						

Continuous variables are expressed as the mean ± standard deviation

*GDM* gestational diabetes mellitus, *BMI* body mass index, *FHR* fetal heart rate, *Bpm* beats per min, *EFW* estimated fetal weight, *HbA1c* glycosylated hemoglobin

P1: GDM (24^+0^–27^+6^ weeks) vs. Controls (24^+0^–27^+6^ weeks)

P2: GDM (28^+0^–40^+0^ weeks) vs. Controls (28^+0^–40^+0^ weeks)

P3: GDM vs. Controls

### Prenatal changes in fetal cardiac morphology in the GDM and control groups

The GDM group showed a significantly lower GSI than the control group during the whole gestational period (Tables 2 and 3). Subgroup analyses according to gestational age (24^+0^–27^+6^ and 28^+0^–40^+0^ weeks) showed that the GSI was still lower in the GDM group than in the control group in each subgroup (Tables 2 and 3). However, there were no differences in GSI between 24^+0^–27^+6^ weeks and 28^+0^–40^+0^ weeks (Tables 2 and 3). Pearson correlation analysis showed little interaction between the GSI and maternal age, BMI, EFW, FHR, and HbA1c (Table 4).

**Table 2:** Comparison of fetal cardiac indices between groups and subgroups

Indices	GDM group (mean ± SD)			Control group (mean ± SD)			*P*-value				
	24^+0^–27^+6^ weeks (*n* = 31)	28^+0^–40^+0^ weeks (*n* = 27)	Total (*n* = 58)	24^+0^–27^+6^ weeks (*n* = 31)	28^+0^–40^+0^ weeks (*n* = 27)	Total (*n* = 58)	P1	P2	P3	P4	P5
4CV-GSI	1.22 ± 0.09	1.19 ± 0.07	1.21 ± 0.08	1.29 ± 0.08	1.26 ± 0.08	1.27 ± 0.08	0.005	0.001	0.000	0.254	0.094
LV-GLS	-18.36% ± 1.11%	-18.14% ± 1.88%	-18.26% ± 1.51%	-22.91% ± 1.39%	-22.46% ± 1.27%	-22.70% ± 1.34%	0.000	0.000	0.000	0.200	0.578
RV-GLS	-18.30% ± 1.27%	-18.77% ± 1.41%	-18.52% ± 1.35%	-23.01% ± 1.55%	-22.43% ± 1.84%	-22.74% ± 1.70%	0.000	0.000	0.000	0.197	0.182
LV-FAC	36.43% ± 4.42%	34.01% ± 4.96%	35.30% ± 4.79%	43.66% ± 5.05%	40.85% ± 4.82%	42.36% ± 5.10%	0.000	0.000	0.000	0.035	0.054
RV-FAC	31.26% ± 4.63%	30.47% ± 4.49%	30.89% ± 4.54%	36.69 ± 2.87%	36.92% ± 3.78%	36.80% ± 3.30%	0.000	0.000	0.000	0.786	0.513

Continuous variables are expressed as the mean ± standard deviation

*GDM* gestational diabetes mellitus, *4CV* four-chamber view, *GSI* global sphericity index, *LV* left ventricle, *GLS* global longitudinal strain, *RV* right ventricle, *FAC* fractional area change

P1: GDM (24^+0^–27^+6^ weeks) vs. Controls (24^+0^–27^+6^ weeks)

P2: GDM (28^+0^–40^+0^ weeks) vs. Controls (28^+0^–40^+0^ weeks)

P3: GDM (total) vs. Controls (total)

P4: Controls (24^+0^–27^+6^ weeks) vs. Controls (28^+0^–40^+0^ weeks)

P5: GDM (24^+0^–27^+6^ weeks) vs. GDM (28^+0^–40^+0^ weeks)

**Table 3:** Adjusted comparison of fetal cardiac indices between groups and subgroups

Indices	Difference between GDM and controls at 24^+0^–27^+6^ weeks		Difference between GDM and controls at 28^+0^–40^+0^ weeks		Difference in controls between 24^+0^–27^+6^ weeks and 28^+0^–40^+0^ weeks		Difference in GDM between 24^+0^–27^+6^ weeks and 28^+0^–40^+0^ weeks	
	Coefficient (95% CI)	*P*-value	Coefficient (95% CI)	*P*-value	Coefficient (95% CI)	*P*-value	Coefficient (95% CI)	*P*-value
4CV-GSI	-0.30 (-0.53, 0)	0.022	-0.44 (-0.65, -0.14)	0.001	-0.02 (-0.27, 0.30)	0.865	-0.24 (-0.51, 0.04)	0..085
LV-GLS	0.87 (0.81, 0.91)	0.000	0.77 (0.64, 0.86)	0.000	0.08 (-0.18, 0.35)	0.579	0.01 (-0.30, 0.36)	0.961
RV-GLS	0.86 (0.79, 0.91)	0.000	0.73 (0.59, 0.83)	0.000	-0.03 (-0.32, 0.30)	0.807	-0.07 (-0.33, 0.21)	0.637
LV-FAC	-0.58 (-0.73, -0.41)	0.000	-0.54 (-0.71, -0.29)	0.000	-0.11 (-0.40, 0.22)	0.434	-0.03 (-0.29, 0.22)	0.855
RV-FAC	-0.57 (-0.75, -0.36)	0.000	-0.63 (-0.80, -0.39)	0.000	0.08 (-0.22, 0.41)	0.555	0.08 (-0.20, 0.38)	0.573

Adjustment made for maternal age, BMI and EFW

*GDM* gestational diabetes mellitus, *4CV* four-chamber view, *GSI* global sphericity index, *LV* left ventricle, *GLS* global longitudinal strain, *RV* right ventricle, *FAC* fractional area change

**Table 4:** Correlations between fetal cardiac indices and variables (Pearson’s correlation coefficients) for all patients

Indices	Maternal age		BMI		EFW		FHR		HbA1c	
	*r*	*P*-value	*r*	*P*-value	*r*	*P*-value	*r*	*P*-value	*r*	*P*-value
4CV-GSI	0.136	0.537	-0.050	0.820	-0.295	0.172	0.192	0.379	0.055	0..804
LV-GLS	-0.012	0.958	0.082	0.709	0.339	0.113	0.321	0.135	-0.199	0.362
RV-GLS	-0.023	0.916	-0.321	0.136	-0.152	0.488	0.167	0.446	-0.163	0.457
LV-FAC	-0.060	0.785	-0.136	0.537	-0.527	0.010	-0.197	0.368	0.378	0.076
RV-FAC	0.134	0.542	-0.079	0.718	0.048	0.829	-0.490	0.018	-0.111	0.613

*BMI* body mass index, *EFW* estimated fetal weight, *FHR* fetal heart rate, *HbA1c* glycosylated hemoglobin, *4CV* four-chamber view, *GSI* global sphericity index, *LV* left ventricle, *GLS* global longitudinal strain, *RV* right ventricle, *FAC* fractional area change

Statistical analyses of the end-diastolic diameter and SI of the LV and RV showed that the 24-segment end-diastolic diameters of the LV and RV were not significantly different between the groups; however, the SIs of 1–6 basal segments of the LV and 5–13 segments of the RV were lower in the GDM group than in the control group (Table 5).

**Table 5:** Comparison of the 24-segment SI between the two groups

24-segment SIs			1	2	3	4	5	6	7	8	9	10	11	12	13	14	15	16	17	18	19	20	21	22	23	24
LV	GDM	Mean	2.22	2.15	2.09	2.04	2.01	2.01	2.02	2.05	2.08	2.12	2.16	2.21	2.27	2.34	2.42	2.51	2.59	2.66	2.77	2.96	3.34	4.13	5.89	11.45
		SD	0.40	0.35	0.31	0.28	0.27	0.26	0.26	0.27	0.28	0.29	0.30	0.32	0.33	0.35	0.37	0.39	0.39	0.38	0.38	0.41	0.49	0.65	0.98	1.95
	Control	Mean	2.04	1.98	1.93	1.89	1.88	1.89	1.92	1.96	2.00	2.05	2.09	2.14	2.20	2.26	2.34	2.43	2.51	2.60	2.70	2.90	3.27	4.04	5.75	11.17
		SD	0.38	0.34	0.31	0.29	0.27	0.27	0.26	0.27	0.28	0.29	0.29	0.31	0.33	0.35	0.38	0.40	0.42	0.43	0.44	0.46	0.52	0.64	0.92	1.79
	*P*-value		0.027	0.020	0.016	0.016	0.019	0.031	0.057	0.105	0.162	0.219	0.250	0.271	0.282	0.291	0.320	0.356	0.397	0.441	0.470	0.491	0.492	0.477	0.470	0.466
RV	GDM	Mean	1.87	1.83	1.78	1.75	1.72	1.70	1.70	1.70	1.72	1.76	1.80	1.86	1.93	2.02	2.12	2.23	2.36	2.51	2.71	3.00	3.48	4.39	6.35	12.42
		SD	0.32	0.30	0.28	0.27	0.26	0.26	0.26	0.26	0.27	0.29	0.30	0.32	0.34	0.37	0.41	0.44	0.47	0.48	0.51	0.56	0.67	0.88	1.32	2.64
	Control	Mean	1.79	1.74	1.69	1.65	1.62	1.60	1.59	1.59	1.61	1.63	1.68	1.73	1.80	1.89	1.99	2.11	2.24	2.40	2.59	2.87	3.34	4.21	6.08	11.88
		SD	0.32	0.29	0.26	0.25	0.24	0.23	0.22	0.22	0.22	0.23	0.23	0.24	0.26	0.28	0.30	0.32	0.35	0.37	0.41	0.47	0.57	0.77	1.15	2.31
	*P*-value		0.198	0.135	0.092	0.064	0.045	0.034	0.027	0.024	0.023	0.024	0.026	0.032	0.043	0.061	0.087	0.122	0.158	0.189	0.219	0.234	0.258	0.277	0.287	0.295

Continuous variables are expressed as the mean ± standard deviation

*GDM* gestational diabetes mellitus, *SI* sphericity index, *LV* left ventricle, *RV* right ventricle

### Prenatal changes in fetal cardiac function in the GDM and control groups

**Fig. 4:**
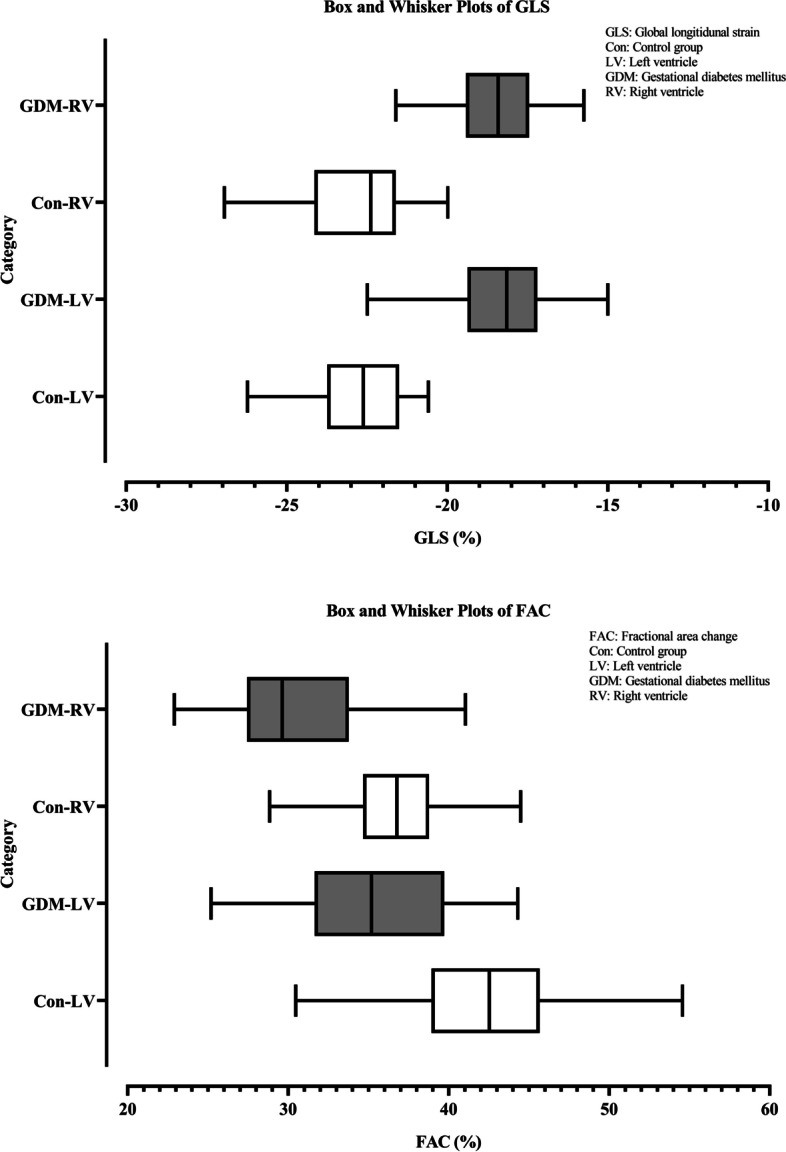
Box plots of GLS and FAC in the GDM and control groups. This figure shows the box plots of GLS and FAC in the GDM group and control group. GLS: global longitudinal strain; FAC: fractional area change; GDM: gestational diabetes mellitus; Cont: control; LV: left ventricle; RV: right ventricle

The GLS of the LV and RV as well as the FAC of the LV and RV were significantly different during the whole gestational period between the GDM and control groups (Tables 2, 3 and Fig. 4). Additionally, the differences in LV-GLS, RV-GLS, LV-FAC, and RV-FAC between the GDM and control groups were retained in subgroup analyses according to gestational age (Tables 2 and 3). Pearson correlation analysis showed a negative correlation between LV-FAC and EFW (Table 4).

### Reproducibility

**Fig. 5:**
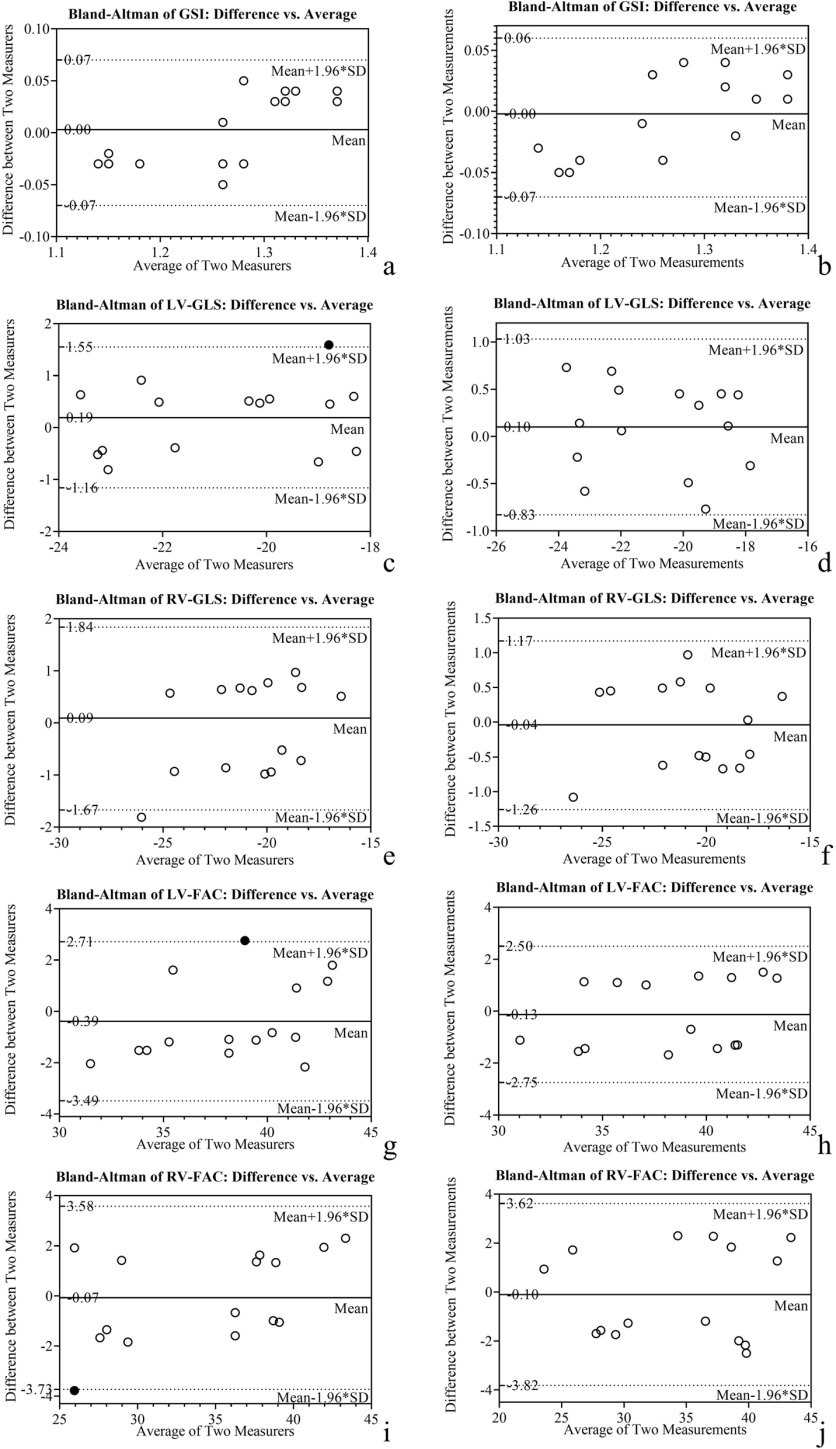
Bland–Altman plots for inter-observer and intra-observer variability. This figure shows the results of inter- and intra-observer variability of GSI, LV-GLS, RV-GLS, LV-FAC, and RV-FAC using Bland–Altman analysis, which demonstrated that the mean variability of inter- and intra-observer results were acceptable. LV: left ventricle; GLS: global longitudinal strain; RV: right ventricle; FAC: fractional area change; GSI: global sphericity index

The ICC were high (> 0.9) for both intra-observer and inter-observer comparisons for all parameters (Table 6). Bland–Altman plots (Fig. 5) showed good agreement between separate measurements of first observer and between the measurements made by two observers.

**Table 6:** Intra-observer and inter-observer correlation

Measurements	Intra-observer		Inter-observer	
	ICC	95% confidence interval	ICC	95% confidence interval
4CV-GSI	0.914	0.763–0.970	0.903	0.737–0.966
LV-GLS	0.975	0.928–0.992	0.941	0.833–0.980
RV-GLS	0.977	0.932–0.992	0.945	0.845–0.981
LV-FAC	0.938	0.826–0.979	0.908	0.749–0.968
RV-FAC	0.957	0.876–0.985	0.953	0.867–0.984

*ICC* intra-class correlation coefficients, *4CV* four-chamber view, *GSI* global sphericity index, *LV* left ventricle, *GLS* global longitudinal strain, *RV* right ventricle, *FAC* fractional area change

## Discussion

Animal studies [4] have shown that exposure to intrauterine hyperglycemia may cause abnormal glucose metabolism in the fetus, which may also alter lipid metabolism and cause excessive production of reactive oxygen species, leading to subsequent oxidative stress [11]. Further, it has been reported [12, 13] that reactive oxygen species can directly damage the systolic function of the heart. Excessive glucose may cause fetal hyperinsulinemia, which may lead to a decrease in fetal blood oxygen levels [14]. Fetal hypoxia is accompanied by a surge in the levels of adrenaline and catecholamines, which cause heart remodeling and hypertrophy, resulting in abnormal fetal cardiac function [4]. In addition, a study involving a rat model showed that maternal hyperglycemia can alter fetal cardiac function by down-regulating the expression of KCNIP2, the key regulatory factor mediating fetal cardiac electrophysiology and contractile function [15].

Although the mechanism by which GDM affects fetal cardiac function is not fully clear, physicians can use prenatal ultrasonography to assess the changes in fetal cardiac function. This prospective cohort study is the first to evaluate the cardiac morphology and function of fetuses of mothers with GDM using the fetal HQ online software. The results showed that GDM had negative effects on fetal cardiac morphology and function starting from the second trimester. We also found that fetal HQ showed good feasibility and repeatability for analyzing fetal cardiac function. Overall, these results support our hypotheses.

### GLS of the LV and RV in the fetuses of mothers with GDM

Strain is a sensitive indicator of cardiac function. According to the stratification and direction of the myocardium, strain can be divided into longitudinal strain, radial strain, and circumferential strain, corresponding to the movement of the myocardium in different directions. Thus, longitudinal strain is mainly used to reflect the movement of the endocardium because of the longitudinal running direction. When ischemia or hypoxia occurs, the endocardium is the first to be damaged, coupled with a reduction in GLS. Thus, an evaluation of the changes in GLS is useful for early diagnosis of impaired cardiac function [16]. Additionally, previous studies [10], which reported results similar to those of our research study, showed stable values of GLS of the LV and RV in normal fetuses and showed no significant relationship of GLS with factors such as maternal age, EFW, and BMI of the mother. In the present study, fetal HQ was used to obtain the GLS values of the LV and RV, which shortened the operative time, improved patient compliance, facilitated operation by the sonographer, and increased feasibility, which highlight the benefits of our research.

Previous studies comparing the GLS of the fetal ventricles between mothers with GDM and a healthy group did not show results consistent with those of our study. Some authors reported that systolic function of the RV was more susceptible to damage than that of the LV. Joana et al. [17] studied 69 pregnant women with GDM and suggested that systolic function was impaired only in the RV in the second trimester. Yovera et al. [18] examined 112 fetuses of women with GDM, including 43 fetuses at a gestational age of 24–32 weeks and 69 fetuses at a gestational age of 32–40 weeks, and reported reduced RV function but normal LV function. The predominance of the RV in the fetal circulation was used to explain the difference between the ventricles. In addition, some authors reported that the GLS values of both ventricles were reduced in the GDM group. Wang et al. [19] analyzed 35 fetuses of mothers with GDM (gestational age: 28–38 weeks) and found that the GLS values of the apical segment of the left ventricular side wall and interventricular septum were reduced. Rolf et al. [6], who examined 53 fetuses of mothers with GDM and 127 fetuses of healthy mothers at a gestational age of 19–39 weeks, found reduced GLS for both the ventricles. The results of Kulkarni et al.’s study [20] also showed that maternal hyperglycemia may cause a reduction in the GLS of the LV in fetuses. Furthermore, the global circumferential strain (GCS) and global radial strain (GRS) were also reduced, indicating an extensive effect on the myocardium, which was similar to the change observed in cardiomyopathy: the nature of the changes in the LV was considered to be the same as that of the changes in the RV. This seems to better explain the unvaried effect of hyperglycemia on the LV and RV in animal models [3]. Another study showed that the reduction in GLS in fetuses of mothers with GDM was retained in the postnatal period [21]. These differences between studies may be related to differences in the diagnostic criteria for GDM and variation between studies in maternal characteristics, diabetes control status, protocol for optimal image acquisition. Besides, van Oostrum [22] reviewed research published in recent years and found a variation in the GLS of both ventricles of normal fetuses, which may be explained by differences in speckle tracking algorithms and ultrasound devices. However, the mechanism underlying the reduction in GLS caused by GDM is still unclear, and further research is needed.

### FAC in the LV and RV in the fetuses of mothers with GDM

FAC is another indicator that reflects the systolic function of the ventricle. Ejection fraction (EF) can only reflect the systolic function of the ventricle, and the systolic function of the LV can be estimated using the two-dimensional Simpson biplane method (four-chamber view or two-chamber view) [23]. This is because the shape of the left ventricular cavity is relatively regular and its cross-section is circular, whereas the cross-section of the RV is crescent-shaped. Therefore, we cannot use the Simpson method to evaluate systolic function of the RV, but instead, FAC can be used to evaluate EF [24].

Yovera et al.’s study [18] found that the FAC in the RV of fetuses of mothers with GDM was significantly reduced at the gestational ages of 24–32 weeks and 32–40 weeks, similar to our results. The systolic function of the RV is mainly attributable to the contraction of longitudinal muscle fibers [25]. Therefore, to a certain extent, the change in FAC may be correlated with the GLS of the RV, which needs to be confirmed by further studies. However, Yovera et al. mentioned that the FAC in the LV in the GDM group only decreased at 24–32 weeks. Besides, there were no differences in the FAC on comparing the two gestational groups, both in the GDM and control groups. These findings are inconsistent with our results. Pearson correlation performed for FAC and other factors indicated that the FAC in the LV had a linear negative correlation with EFW (Table 4), while the FAC in the RV had no obvious correlation with EFW. Such differences may be caused by differences in grouping and the distribution of gestational age or differences in the rate of growth of the ventricles. DeVore et al. [26] found that at the gestational age of 20–30 weeks, the FAC in both ventricles decreased slowly with increasing gestational age and then remained stable. Further large-sample multicenter studies on the alterations in FAC in healthy fetuses and the influence of GDM on FAC are required.

### GSI and 24-segment SI in the fetuses of mothers with GDM

Previous studies have found that the shape and size of the heart were closely related to the structure and function of the heart [27, 28]. As an emerging technology, fetal HQ integrates STE and 24-segment myocardial analysis, assessing cardiac function in a more detailed manner. In addition to the GSI, the 24-segment SI can be analyzed, which is another highlight of our research. The GSI of the heart, the ratio of the overall longitudinal length to the transverse length of the 4CV at end-diastole, can be used to assess the overall shape of the heart. Moreover, the 24-segment SI of the ventricles, the ratio of the longitudinal length to the transverse length in each segment of the ventricle, can be used to evaluate the shape of each ventricle. Previous studies have found that there was no significant correlation between the 24-segment SI and fetal size or gestational age [27], which has been confirmed by our research, and this finding laid the foundation for evaluating the changes in the shape of the heart.

The structure of the heart is complex, including the four chambers macroscopically. The whole heart can look more coordinated only if it maintains a relatively fixed ratio between all of its parts. A change in the shape or size of a certain part does not necessarily affect the overall shape or size of the heart. Thus, even if the overall shape and size seem to be normal, it is still necessary to measure the shape and size of each chamber when the proportion of one part seems to be abnormal [27].

According to our study, the GSI was relatively stable and did not change with increasing gestational age in the control group. Additionally, the GSI of fetuses of mothers with GDM was slightly lower than that of healthy fetuses starting from the second trimester, which indicated that the fetal heart had a rounder shape in the GDM group than in the control group. This difference may be related to fetal hypoxia [29]. There was no significant difference between the GDM and control groups in the analysis of the 24-segment end-diastolic diameters of each ventricle. However, there was a significant difference in the SI of the basal segments of the LV in the analysis of the 24-segment SI. We speculated that this difference may be related to the shape of the LV itself and the effect of hyperglycemia on the myocardium. According to the data of the control group, the SI of the RV was significantly lower than that of the LV for segments 1–19, suggesting that the RV has a more globular shape at the basal, mid, and proximal apical segments. This finding is roughly consistent with those of a previous study [27]. The difference in the SI indicated that the normal anatomical structure of the LV had a bullet shape, whereas that of the RV had a pyramidal shape. Therefore, small changes in the shape of the LV were more likely to cause changes in the SI than those in the shape of the RV. This does not mean that the decrease in the GSI was only caused by the abnormal SI of the segments of the LV, but it should be understood that a “quantitative change caused a qualitative change”.

### Strength and limitations

Fetal HQ is a new technology and can be considered as a fusion of TomTec’s Cardiac Performance Analysis and GE Voluson E10 Ultrasound System. In addition to the aforementioned advantages, this technology seems to be more suitable for the evaluation of the fetal heart compared to previously used methods (applying the adult mode that divided the LV into 16 or 17 segments according to the coronary blood flow distribution). Besides, Fetal HQ has a lower operating threshold compared with other speckle tracking software. 4CV, which is a necessary section of fetal cardiac ultrasound examination, is also the easiest view to obtain. However, the speckle tracking software of other manufacturers needs to obtain the “three-chamber heart” view and “two-chamber heart” view except 4CV. Due to the changeable position, bones, and depth of fetus, it sometimes becomes impossible to obtain these three views at the same time, which greatly increases the threshold of the analysis. What’s more, the evaluation of fetal cardiac function using Fetal HQ is faster, which can be reflected in two aspects. One is that the preparation time before analysis, that is, the image acquisition time is short—the time to obtain a 4CV image is much shorter than the three views combined. The other is the short time of analysis. Fetal HQ does not require offline analysis or layered analysis, which shortens part of time. Finally, in addition to providing cardiac functional parameters like GLS, Fetal HQ also can evaluate changes in cardiac morphology, such as SIs.

However, this study has several limitations. First, this was a single-center study, and the gestational ages of the study subjects were not evenly distributed, with few pregnant women at a gestational age ≥ 36 weeks. Secondly, the design was not longitudinal. Many of the pregnant women would not go back to the following-up echo as they may live in the suburbs or other cities. Moreover, most of the pregnant women with GDM in our study showed good blood sugar control; thus, we could not obtain results for fetuses of mothers with poor blood sugar control. Future studies still need to supplement the effects of glycemia, anti-diabetic therapy and its duration on GLS, FAC, GSI, 24-segment SI, and other factors. Additionally, the long-term follow-up after birth should also be improved to observe the influence of the changes in the indicators on the prognosis.

## Conclusions

In conclusion, fetal HQ can easily and quickly assess fetal cardiac morphology and function. The GLS, FAC, and GSI were reduced in the fetuses of mothers with GDM, starting from the second trimester. These changes may be related to remodeling of the myocardial structure and impairment in cardiac function caused by hyperglycemia, which indicated that the effects of GDM on fetal cardiac morphology and function appeared early. Thus, whether earlier and stricter clinical intervention is necessary remains to be further studied. Furthermore, the underlying mechanism by which hyperglycemia affects cardiac function is still unclear, and further research is needed.

## Supplementary Information

**Additional file 1:** **Video 1**. The results of speckle-tracking analysis. Information regarding the GLS and FAC of the LV and RV and the movement trajectory of the LV and RV can be obtained from the result interface. GLS: global longitudinal strain; FAC: fractional area change; LV: left ventricle: RV: right ventricle

## Data Availability

The datasets supporting the conclusions of this article are included within the manuscript (and its additional files). The authors would like to share raw anonymized video data related to the current study, which could only be used for personal study. The demanders may contact baogoubei@hotmail.com.

## Abbreviations

AC: Abdominal circumference
BMI: Body mass index
BPD: Biparietal diameter
EF: Ejection fraction
EFW: Estimated fetal weight
FAC: Fractional area change
Fetal HQ: Automatic Fetal Heart Assessment Tool
FHR: Fetal heart rate
FL: Femur length
GCS: Global circumferential strain
GDM: Gestational diabetes mellitus
GLS: Global longitudinal strain
GRS: Global radial strain
GSI: Global sphericity index
HbA1c: Glycosylated hemoglobin
HC: Head circumference
ICC: Intra-class correlation coefficients
LV: Left ventricle
OGTT: Oral glucose tolerance test
RV: Right ventricle
SD: Standard deviation
SI: Spherical index
STE: Speckle-tracking echocardiography
4CV: The four-chamber view
